# Patterns of interaction specificity of fungus-growing termites and *Termitomyces *symbionts in South Africa

**DOI:** 10.1186/1471-2148-7-115

**Published:** 2007-07-13

**Authors:** Duur K Aanen, Vera ID Ros, Henrik H de Fine Licht, Jannette Mitchell, Z Wilhelm de Beer, Bernard Slippers, Corinne Rouland-LeFèvre, Jacobus J Boomsma

**Affiliations:** 1 Laboratory of Genetics, Plant Sciences Group, Wageningen University and Research Center, Arboretumlaan 4, 6703 BD Wageningen, The Netherlands; 2 Department of Population Biology, Institute of Biology, University of Copenhagen, Universitetsparken 15, 2100 Copenhagen, Denmark; 3 Agricultural Research Council-Plant Protection Research Institute, Rietondale Research Station, Private Bag X134, Queenswood, Pretoria 0121, South Africa; 4 Forestry and Agricultural Biotechnology Institute (FABI), Faculty of Agricultural and Biological Sciences, Department of Microbiology and Plant Pathology, University of Pretoria, Pretoria, South Africa; 5 UMR-IRD 137 Biosol Laboratory of Tropical Soils Ecology (LEST) – Centre IRD d'Ile de France, 32 avenue Henri Varagnat 93 143 – Bondy Cedex, France; 6 Evolutionary Biology, Institutefor Biodiversity and Ecosystem Dynamics, University of Amsterdam, P.O. Box 94062, 1090 GB Amsterdam, The Netherlands

## Abstract

**Background:**

Termites of the subfamily Macrotermitinae live in a mutualistic symbiosis with basidiomycete fungi of the genus *Termitomyces*. Here, we explored interaction specificity in fungus-growing termites using samples from 101 colonies in South-Africa and Senegal, belonging to eight species divided over three genera. Knowledge of interaction specificity is important to test the hypothesis that inhabitants (symbionts) are taxonomically less diverse than 'exhabitants' (hosts) and to test the hypothesis that transmission mode is an important determinant for interaction specificity.

**Results:**

Analysis of Molecular Variance among symbiont ITS sequences across termite hosts at three hierarchical levels showed that 47 % of the variation occurred between genera, 18 % between species, and the remaining 35 % between colonies within species. Different patterns of specificity were evident. High mutual specificity was found for the single *Macrotermes *species studied, as *M. natalensis *was associated with a single unique fungal haplotype. The three species of the genus *Odontotermes *showed low symbiont specificity: they were all associated with a genetically diverse set of fungal symbionts, but their fungal symbionts showed some host specificity, as none of the fungal haplotypes were shared between the studied *Odontotermes *species. Finally, bilaterally low specificity was found for the four tentatively recognized species of the genus *Microtermes*, which shared and apparently freely exchanged a common pool of divergent fungal symbionts.

**Conclusion:**

Interaction specificity was high at the genus level and generally much lower at the species level. A comparison of the observed diversity among fungal symbionts with the diversity among termite hosts, indicated that the fungal symbiont does not follow the general pattern of an endosymbiont, as we found either similar diversity at both sides or higher diversity in the symbiont. Our results further challenge the hypothesis that transmission-mode is a general key-determinant of interaction specificity in fungus-growing termites.

## Background

Mutualistic interactions between species are common and have played a central role in the diversification of life [1]. Interactions range from temporal, facultative encounters to obligate permanent symbioses. Mutualistic symbioses also often represent major and ecologically highly successful transitions in evolution [2]. Co-evolution of mutualistic taxa involves reciprocal evolutionary change through natural selection [3]. If co-evolutionary interactions persist through speciation events, whole clades of different species can co-speciate [4]. However, co-cladogenesis is only an extreme outcome of coevolution as specificity can also exist at other levels than the species level. For example, when symbionts regularly switch between different host species, they may still be specific to a particular host genus.

**Figure 1:**
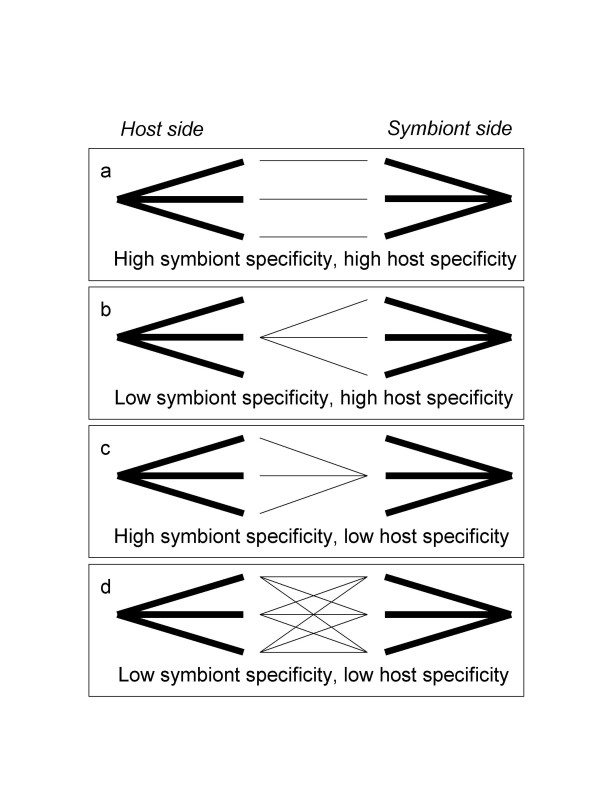
The theoretically possible patterns of specificity in a mutualistic host-symbiont interaction. a. Mutually high specificity. One host lineage is exclusively associated with a single symbiont lineage. b. Low symbiont specificity, high host specificity. A host lineage can be associated with three different symbiont lineages (low symbiont specificity), which each are specialized on that single host lineage (high host specificity). c. High symbiont specificity, low host specificity. Three different host lineages are each associated with the same symbiont lineage. d. Mutually low specificity. Three host lineages can all be associated with three different symbiont lineages.

Specificity in symbiotic interactions has two sides, the host and the symbiont. From the symbiont perspective of specificity, usually the term 'host specificity' is used, which is defined on the basis of the range of hosts that a symbiont can utilize as a partner. Analogously, from the host perspective of specificity we introduce the term 'symbiont specificity' which is defined on the basis of the range of symbionts that a host can utilize. In Figure 1, the different possibilities are summarized. As an umbrella term for the different types of specificity we use the general term 'interaction specificity'.

Numerous factors have been hypothesized to influence interaction specificity. For example, Law and Lewis [5] noticed that the taxonomic diversity and the frequency of sex are generally lower in the 'inhabitant' (the 'symbiont') than in the 'exhabitant' (the 'host') of mutualistic interactions. Another important variable for patterns of specificity is the transmission mode of symbionts. Vertical transmission greatly limits the frequency by which new combinations of symbiotic partners arise and may therefore lead to a higher degree of coevolution than horizontal transmission, although many exceptions to this prediction have been found [6].

The fungus-growing social insects are interesting and ecologically important examples of mutualistic symbiosis with variable degrees of specificity. Growing fungi for food has independently evolved in the old-world macrotermitine termites (c.f. [7-9]) and in the new-world attine ants (c.f. [10,11]). Interaction specificity and co-evolution have been studied intensively in the fungus-growing ants [*e.g*. [10-12]], but much less is known about the fungus-growing termites. However, the opportunities provided by the fungus-growing termites and their *Termitomyces *symbionts to obtain insight in the co-evolution of interaction specificity are substantial because the transmission modes and reproductive systems of the termite symbionts vary much more than those of the fungus-growing ants [9,13,14].

### Natural history of fungus-growing termites

The fungus-growing termites have evolved into approximately 330 extant species, belonging to ca. 12 genera [15]. The termites maintain their fungal symbiont on special structures in the nest, the fungus combs, which are housed in specially constructed chambers, either inside a mound or dispersed in the soil. Workers feeding on dry plant material produce fecal pellets (*primary feces*) which are added continuously to the top of the comb and fungal mycelium rapidly develops in the newly added substrate. After a few weeks, the fungus starts to produce vegetative structures, *nodules *(which are modified unripe mushrooms) that are consumed by the termite workers. At a later stage, the entire comb structure permeated with mycelium is consumed [8].

Direct and indirect evidence indicates that symbiont transmission between colonies and across generations is normally sexual and horizontal [[16-20], for a review, see [21]]. This implies that new colonies will usually start their existence without a fungus. The most likely route to acquire the fungal symbiont is via sexual spores produced by mushrooms from other nests. The single study performed so far on the genetic population structure of the fungal symbiont of the species *M. natalensis *[18] has shown a freely-recombining population structure of the fungus, which is consistent with sexual horizontal transmission as the main transmission mode. At least two independent transitions to clonal, vertical and uniparental transmission have occurred [9]: one involving a single species, *Macrotermes bellicosus *(via the male sexuals; [17]) and one possibly involving the entire genus *Microtermes *(via the female sexuals; based on the five studied species; [17,22]). With vertical transmission, sexuals of one of the two sexes ingest asexual spores before the nuptial flight and use these as inoculum for the new fungus comb after colony foundation.

The symbiosis between termites and *Termitomyces *fungi is 'symmetric' since both partners are obligatorily interdependent, and this dependence has a single evolutionary origin with no known reversals to non-symbiotic states [9,23,24]. Furthermore, specificity in interactions occurs at higher taxonomic levels, i.e. (combinations of) genera tend to rear different clades of *Termitomyces *[9]. This higher-level specificity strongly suggests that coevolution between termites and fungi, has occurred. Within genera, however, generally no strong association between the evolutionary histories of the termites and their fungal symbionts has been found [9]. At the species level, opposite specificity patterns have been observed. In some cases, the sampled fungal symbionts of a single termite species did not form a monophyletic group (*e.g*. the fungal symbionts of *Macrotermes bellicosus *and *Odontotermes latericius*) [9], but the opposite pattern, where the termite hosts of a single symbiont did not form a monophyletic group, has also been found. An extreme example of the latter was provided by the single fungal lineage shared between all sampled colonies in western Africa of the three divergent genera *Microtermes*, *Ancistrotermes *and *Synacanthotermes *[9]. Recently, it was found that *Macrotermes natalensis *has a very specific association as it was exclusively associated with a single fungal lineage [18], showing that strong specificity can also exist at the species level.

These conflicting patterns of interaction specificity indicate that further work is necessary before any general conclusions about interaction specificity at lower taxonomic levels can be drawn. The present paper offers a detailed comparative study of the interaction specificity of South African fungus-growing termites. We investigated how specific the interactions between termites and fungal symbionts in South Africa are at the genus, species and population level and how the different levels of specificity might relate to inferred co-evolutionary dynamics and known modes of transmission. To address these questions we obtained sequence data from 101 colonies belonging to eight species in three genera of South African fungus-growing termites and estimated interaction specificity at the genus, species and colony level.

## Results

### Sequence analyses

**Figure 2:**
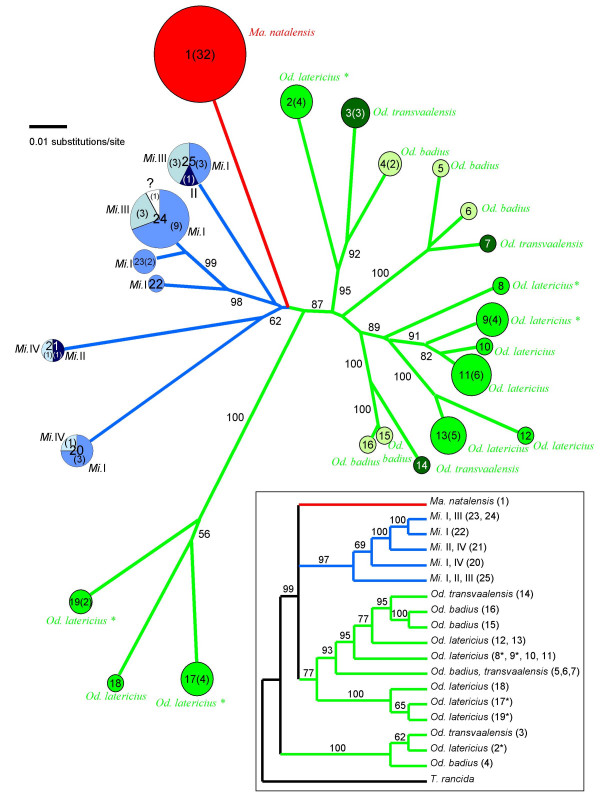
**a**. Unrooted neighbor-joining phylogram of the 25 ITS haplotypes found among the *Termitomyces *symbionts of 101 nests of fungus growing termites (belonging to eight species in three genera). The three genera are indicated with different colours, and the species within the genera in different intensities of these colours. The Senegalese samples of *O. latericius *are indicated with an asterisk. Within each circle the haplotype number is indicated and, in brackets, the number of that haplotype found (if more than one). The area of circles is proportional to the observed frequency of particular haplotypes. Red: genus *Macrotermes*; green: genus *Odontotermes*; blue: genus *Microtermes*. Within the genus *Microtermes *four tentative species were distinguished (labelled I-IV). For one *Microtermes *termite sample no sequence was obtained, which is indicated with a '?'. The numbers on the branches are the percentage bootstrap values (> 50) based on 1000 bootstrap replicates. **b. **Phylogenetic relationships between the *Termitomyces *symbionts of the fungus growing termites included in this study. The cladogram is the majority rule consensus tree of trees sampled in a Bayesian analysis of combined partial nuclear 25S sequences and mitochondrial 12S sequences. The free-living fungus *Tephrocybe rancida *was used as an outgroup, based on previous analyses (Hoffstetter *et al*. 2002; Aanen *et al*., 2002). Abbreviations used: *Ma*.: *Macrotermes*; *Mi*.: *Microtermes*; *Od*.: *Odontotermes*.

Complete ITS sequences were obtained for 101 strains (Table 1; Table 1 in Additional file 1) and sequence length ranged from 597 to 690 nucleotides. Of the 794 positions in the final alignment, 300 were variable and 179 of these were parsimony informative. Gap positions were coded as missing data. In Figure 2a the unrooted neighbor-joining phylogram is given, using uncorrected pairwise distances as a distance measure. Using MAFFT [25] to align the sequences instead of ClustalW resulted in an almost identical neighbor joining topology (not shown).

**Table 1:** The termite samples collected at the different locations and the associated genetic diversity of their *Termitomyces *symbionts (See Figure 1 for further details)

Termite species	site	number of samples	ITS haplotypes
*Macrotermes natalensis*	ZA1	1	1
	ZA3	14	1
	ZA5	3	1
	ZA6	1	1
	ZA7	9	1
	ZA8	3	1
	ZA9	1	1
*Odontotermes badius*	ZA3	5	4, 5, 6, 15, 16
	ZA5	1	4
*Odontotermes latericius*	ZA3	14	10, 11, 12, 13, 18
	ZA4	1	10
	Se1	8	2, 8, 9, 17, 19
	Se2	6	2, 17
*Odontotermes transvaalensis*	ZA5	1	7
	ZA4	4	3, 14
*Microtermes sp. I*	ZA2	1	24
	ZA3	14	20, 22, 23, 24, 25
	ZA5	1	20
	ZA9	1	25
	ZA10	1	24
*Microtermes sp. II*	ZA7	2	21, 25
*Microtermes sp. III*	ZA4	1	24
	ZA5	1	24
	ZA7	4	24, 25
*Microtermes sp. IV*	ZA5	2	20, 21
*Microtermes?*	ZA3	1	24

Genbank accession numbers for fungal ITS, nuclear 25S, mitochiondrial 12S and the COI sequences of *Microtermes *sp. I, II, III and IV are given in the online supplementary table.

A rooted phylogeny was estimated in a second analysis, using the more conserved nuclear 25S and the mitochondrial 12S on a single representative of each ITS haplotype, while using the non-symbiotic fungus *Tephrocybe rancida *as an outgroup [9,24,26]. In Figure 2b the majority rule consensus tree of the trees sampled in the Bayesian analysis is given. Maximum parsimony and neighbour-joining analyses gave similar results.

Within the sampled termites of the genus *Microtermes*, four main COI lineages were found. We tentatively consider these to represent different species (designated as I, II, III and IV).

### Levels of interaction specificity

Analysis of Molecular Variance of the fungal sequence variation across various taxonomic levels showed that on average 47 % of the symbiont sequence variation occurred between termite genera, 18 % between termite species, and 35 % between colonies within species (Table 2). High bilateral specificity existed at the genus level, as no symbiont lineages were shared between the three genera *Macrotermes*, *Microtermes *and *Odontotermes *and the symbionts of these genera formed three separate clusters in the phylogram of the aligned ITS sequences (indicated with different colours; Figure 2a). In the rooted analysis (Figure 2b) the symbionts of the genera *Macrotermes *and *Microtermes *formed two monophyletic groups, while the symbionts of *Odontotermes *consisted of two distinct non-sister clades.

**Table 2:** Results of AMOVA of *Termitomyces *symbionts at three hierarchical levels.

***Source of variation***	*d.f*.	*Sum of squares*	*Variance component*	*% variation*	*Fixation indices*	*P value*
**Between genera**	2	2064.85	28.72	47	Φ_CT _= 0.47	0.0029
**Among species within genera**	5	439.53	11.18	18	Φ_SC _= 0.34	0.0020
**Within species**	77	1677.90	21.79	35	Φ_ST _= 0.65	< 0.0001
**Total**	**84**	**4182.27**	**61.68**	**100**		
***Within Odontotermes: Φ***_*ST*_*** = 0.25 (p < 0.001)***						
***Comparison ***	*Pairwise Φ*_*ST*_		*P value*			
***latericius & transvaalensis***	0.34		p < 0.001			
***latericius & badius***	0.23		p = 0.002			
***transvaalensis & badius***	0.08		p = 0.19; n.s.			
***Within Microtermes: Φ***_*ST*_*** = 0.13 (p = 0.08)***			0.37			
***Comparison ***	*Pairwise Φ*_*ST*_		*P value*			
***Sp. I & sp. II***	0.21		p = 0.11; n.s.			
***Sp. I & sp. III***	0.01		p = 0.58; n.s.			
***Sp. I & sp. IV***	0.27		p = 0.13; n.s.			
***Sp. II & sp. III***	0.10		p = 0.36; n.s.			
***Sp. II & sp. IV***	-0.36		p = 0.99; n.s.			
***Sp. III & sp. IV***	0.37		P = 0.04			
***Sp. I & sp. III***	0.01		p = 0.58; n.s.			
***Sp. I & sp. IV***	0.27		p = 0.13; n.s.			
***Sp. II & sp. III***	0.10		p = 0.36; n.s.			
***Sp. II & sp. IV***	-0.36		p = 0.99; n.s.			
***Sp. III & sp. IV***	0.37		P = 0.04			

Specificity was generally low at the level of termite species, with the exception of *Macrotermes natalensis *where all 31 sampled mounds had the same unique ITS lineage of *Termitomyces *[18]. However, since *Macrotermes natalensis *was the only species sampled in this genus, we could not determine whether the ITS lineage that we found is specific for this particular *Macrotermes *species. The three species in the genus *Odontotermes *were all associated with a highly diverse set of fungal symbionts, which did not form a monophyletic group for any of these species (Figure 2b). However, AMOVA showed that significant differences occurred between the species (Table 2). Pairwise comparisons revealed that these differences were mainly due to the symbionts of *O. latericius *being different from the symbionts of the two other *Odontotermes *species, *O. badius *and *O. transvaalensis *(the symbionts of *O. badius *and *O. transvaalensis *were not significantly different: P = 0.19). Interestingly, although the three *Odontotermes *species were all associated with a wide range of fungal symbionts, none of the ITS haplotypes were shared between species. To test the significance of this result, we also made pairwise comparisons between the *Odontotermes *species, using only the frequency of the different haplotypes within species, and not the actual sequence information [27,28]. This revealed that the differentiation of fungal symbionts between species was indeed significant for all three pair-wise comparisons. However, this result may be due to our sampling effort: a high overall diversity of *Odontotermes*-associated *Termitomyces *in combination with spatial autocorrelation may explain this result. In line with this, only two haplotypes (4 and 10) were found at more than one locality.

A different pattern was found for the variation in *Termitomyces *symbionts between species of the genus *Microtermes*, as most of the six fungal haplotypes were shared between species (Figure 2a) and there were no significant differences between species (p = 0.08; Table 2).

## Discussion

Grassé [29] proposed that every genus of fungus-growing termites was associated with a single species of *Termitomyces*. However, it was recently shown [9,24] that this hypothesis is invalid and that different morphospecies of *Termitomyces *can be associated with the same genus of termites and sometimes with single species. Here, we detail this further by showing that many species of fungus-growing termites are associated with highly divergent strains of *Termitomyces *that probably represent different morphospecies. The symbiont diversity that we have found in *Odontotermes *and *Microtermes *species is the highest reported for any fungus-growing termite so far. Compared to the results reported here, Katoh *et al*. [30] found only minor intraspecific symbiont diversity (two ITS haplotypes) in SE Asian *Odontotermes formosanus*. This is consistent with Africa being the core area where the association between termites and fungi arose and radiated, whereas SE Asia represents the edge of the present distribution of this symbiosis [31].

### Interpreting differences in interaction specificity

Genus-level symbiont specificity was high, as symbiont lineages formed completely separate clusters without any overlap. This is consistent with the patterns of specificity above the species level that were documented previously [9]. At the species level, however, we found differences in interaction specificity between the three genera: specificity was high for both sides in *Macrotermes natalensis *(situation a in figure 1) and much lower in the other two genera. Although AMOVA showed that significant differences occurred between species within the genus *Odontotermes*, pairwise comparisons revealed that this difference was due to the symbionts of *O. latericius *being different from the symbionts of the two other *Odontotermes *species, *O. badius *and *O. transvaalensis*, which themselves were not significantly different. Nevertheless, none of the ITS haplotypes were shared between these three *Odontotermes *species, and the overall differentiation between species was significant. One possible interpretation of this result is that symbionts are exchanged between *Odontotermes *species, because each species would otherwise be associated with a monophyletic group of symbionts, but that this exchange is rare enough to allow the evolution of some differentiation between species. However, an alternative explanation is that this result is a sampling artifact: a high overall diversity of *Odontotermes*-associated *Termitomyces *in combination with spatial autocorrelation may explain this result. In line with this interpretation, only two haplotypes (4 and 10) were found at more than one locality.

Low specificity at both sides was found for the studied species of the genus *Microtermes*, which essentially shared a common pool of genetically diverse symbionts (situation d in figure 1). A completely different pattern has previously been found in species of the genera *Microtermes*, *Ancistrotermes *and *Synacanthotermes *collected in western Africa (Senegal and Cameroon), which were all associated with a single fungal lineage with little sequence divergence (consistent with situation c in figure 1; [9]. The reason for this difference in *Microtermes *symbiont diversity between the two African regions is presently unclear, but the observed patterns do suggests that specificity and co-evolutionary dynamics may differ both geographically and between closely related termite species, as envisaged in geographic mosaic models for co-evolution [1].

### Are the observed patterns consistent with the Law and Lewis hypothesis?

Law and Lewis [5] argued that the exhabitant (host) in a mutualistic symbiosis is generally more diverse and has a higher frequency of sex than the inhabitant(s) (the symbiont(s)). They hypothesized that different host species may converge, evolutionarily, on a single mutualist symbiont, whereas they will disperse in phenotypic space to escape the attacks of a parasite, thus encouraging speciation in the latter [2]. The question is whether this inhabitant-exhabitant distinction is equally valid for the symbiosis between Macrotermitidae and *Termitomyces*, which is an ectosymbiosis relative to the individual termites and an endosymbiosis (*sensu* Law and Lewis) only for the entire colony of termites. The patterns that we found are not consistent with the Law and Lewis predictions for most of the species of fungus-growing termites that we studied, because they tend to be associated with a genetically highly diverse set of symbionts. Thus, although the fungus-growing termites create a benign environment for the fungus inside their nest, the fungus garden may not be as well protected from external parasitic microorganisms as a real endosymbiont would be, so that selection for symbiont genetic diversity at the population level is maintained in most groups of fungus-growing termites. This is also consistent with the high frequency of sex in most of the *Termitomyces *symbionts [18,21]. Interestingly, a similar pattern of high genetic diversity at the population level has been found among the symbionts of fungus-growing ants [12,32,33], but here the frequency of sex among symbionts is apparently much lower [34].

### Is interaction specificity linked to transmission mode?

Symbiont transmission modes have been studied for a rather limited number of macrotermitine species. For the species included in this study, we have direct evidence that *Odontotermes badius *and *Macrotermes natalensis *rely on horizontal symbiont acquisition [18,19]; De Fine Licht, Korb and Aanen, pers. obs.). For the other two included species of *Odontotermes *we have indirect evidence, because: 1. fruiting occurs frequently for both species; 2. all other species of the genus that have so far been tested have horizontal symbiont acquisition [17,20]. Also our inferences for *Microtermes *have to be based on indirect evidence: fruiting bodies have never been observed for any of the species included in the present study and five other species of this genus are known to rely on vertical, uniparental symbiont transmission via the female sex [17,22].

Unexpectedly, the presumably vertically transmitted fungi of the three *Microtermes *species were shared between species. This indicates that horizontal transmission must occur frequently enough to prevent any host specificity at the species level. In an earlier study [9] it was found that the fungal symbionts of several west African *Microtermes *species were identical to the symbionts of the divergent genera *Ancistrotermes *and *Synacanthotermes*, showing that horizontal transmission also occurred between these genera in this other region. Obviously, the transmission modes for these species need to be addressed directly in future studies.

### How does interaction specificity arise?

For the termite species with horizontal symbiont acquisition, it is still an open question how combinations between termites and their fungal symbionts arise and how specificity arises. One way in which interaction specificity could arise would be geographical isolation, but we did not find strong geographical differentiation at the scale of our study. For some species of fungus-growing termites it has been found that fungal fruiting is synchronized with the time that the first workers of founding colonies of the typical host leave the nest to start foraging [17]. Since dispersal on the wing varies between species, but is highly synchronized within species, such temporal segregation would be a possible way for interaction specificity to arise. Temporal segregation of this kind could potentially explain that all three *Odontotermes *species in our study were associated with a different range of fungal symbionts, so that none of the ITS haplotypes were shared between the species. A related question of crucial importance is how long basidiospores of *Termitomyces *remain viable and whether a functional *Termitomyces *spore bank exists. We hypothesize that active partner selection may also play a role in the establishment of interaction specificity, as neither temporal nor geographical isolation seem sufficient explanations to arrive at the levels of specificity observed. Similarly, for species with predominantly vertical transmission, the question is how symbiont exchange occurs. Detailed experimental studies will be needed to investigate whether and how selection takes place during the establishment and later maintenance of fungal symbionts in termite colonies.

## Conclusion

Interaction specificity between fungus-growing termites and *Termitomyces *fungi is highly variable and ranges from mutually high specificity to mutually low specificity (Figure 1). This implies that the hypothesis that inhabitant mutualistic symbionts should be less diverse than the exhabitant hosts is not supported by the data, as we found either similar diversity at both sides or higher diversity in the symbiont. Our results further challenge the hypothesis that transmission-mode is a general key-determinant of interaction specificity in fungus-growing termites.

## Methods

### Samples and identification of termites

**Figure 3:**
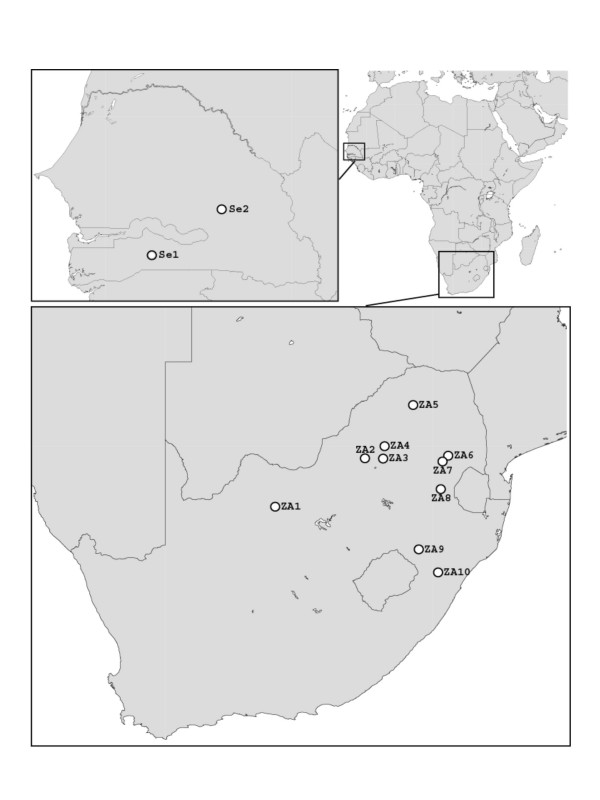
Sampling localities in Senegal and the Republic of South Africa. Abbreviations used: Se1 = Thiès, Senegal; Se2 = Kolda, Senegal; Se3 = Tambacounda, Senegal; ZA1 = Kalahari (S27.10 E24.11); ZA2 = Pretoria surroundings (S25.42 E27.47); ZA3 = Pretoria PPRI Rietondale (S25.43 E28.14); ZA4 = SABS Farm, Radium, South Africa (S25.00 E28.21); ZA5 = Pietersburg (S29.27 E23.54); ZA6 = Sabie, South Africa (S25.33 E30.58); ZA7 = Vygeboomdam, South Africa (S25.53 E30.37); ZA8 = Amsterdam surroundings, South Africa (S26.50 E30.30); ZA9 = Kwazulu Natal, South Africa (S28.57 E29.48); ZA10 = Pietermaritzburg, South Africa (S29.35 E30.20).

Eighty six nests of fungus-growing termites, belonging to three genera and eight species were sampled in South Africa in January 2003 and January 2004 (Figure 3; Table 1; Table 1 in Additional File 1). Several worker and soldier termites and some comb material were preserved in 96% alcohol for each nest, and stored at 4°C for future reference or DNA extraction. The fungus of most colonies was isolated in pure culture (Table 1 in Additional File 1) from nodules on malt yeast extract agar (per liter: 20 g malt extract, 2 g yeast extract and 15 g agar). For one species, *Odontotermes latericius*, 15 additional samples were obtained from four sites in Senegal in December 2000. Here, only termites were sampled by collecting > 10 workers from foraging trails and storing them in 96% alcohol. Fungal sequences were obtained from the guts of these termites using *Termitomyces *specific primers (see below). The distance between foraging trails was at least 10 meters so that the chance of sampling the same nest twice was low. Nonetheless, in cases where multiple identical sequences were found within a site, only one of these was included in the analysis.

Most termite samples of the genera *Macrotermes *and *Odontotermes *could be identified at the species level on the basis of morphological characters. However, the taxonomy of the genus *Microtermes *is complex and awaits further study [35] so that the *Microtermes *samples could not be identified. In order to classify the *Microtermes *samples in "species groups", we sequenced a ca. 1000 bp region of the mitochondrial cytochrome oxydase I (COI) gene, using only the forward primer as a sequencing primer (resulting in a readable sequence of at least 500 but usually more than 800 nucleotides). Sequences that differed less than 4% were considered to belong to the same tentative species. PCR amplifications were done using previously published primers (forward: BL1834, 5'TCAACAAATCATAAAGATATTGG3' or TL1862, 5'TACTTCGTATTCGGAGCTTGA3' and reverse: a non-degenerate version of TH2877, 5'TAGGTGTCGTGTAATACAATGTC3'; [9,31]. Sequences of representatives of the recognised tentative species have been submitted to Genbank (accession numbers in Table 1 in Additional File 1).

### Fungal sequences and phylogenetic analyses

The analysis of the *Termitomyces *diversity in our samples consisted of two steps. First, a hierarchical analysis of interaction specificity was done using the highly variable ITS sequences. Second, the phylogenetic position of the different ITS haplotypes was determined by using more conserved DNA regions and including an outgroup. The high variability of the ITS region made alignment ambiguous and less suitable for estimating higher-order relationships. We therefore refrained from using the ITS sequences to make phylogenetic inferences and used two more conserved regions of nuclear 25S rDNA and mitochondrial 12S RNA instead (details given below).

For all 101 samples, the nuclear ribosomal region including ITS1, 5.8S, and ITS2 was amplified and sequenced using the primers ITS1 and ITS4 [36]). ITS sequences for *M. natalensis *were determined in a previous study [18] and included in the present analysis. In Senegal we had only sampled termites, so that we obtained total fungal DNA from termite abdomens (including gut contents; see [9]), and amplified ITS sequences by using a *Termitomyces *specific primer (ITS1FT: GTTTTCAACCACCTGTGCAC, based on available sequences in GenBank), which was used in combination with the universal primer ITS4 [36]). Sequencing was done using the forward and backward primer, and in some cases the internal primers ITS2 and ITS3 [36]). For some strains, two ITS copies were present in the PCR products, differing by a small length mutation, giving chromatograms with double peaks for part of the sequence. However, using both forward and reverse primers and sometimes internal primers, the two copies could be recovered. Of these, we selected a single sequence for the analysis, so that our data are effectively haplotypic. Selection of the other copy did not affect the results (data not shown). Sequences were aligned using ClustalW [37]; settings for both pairwise alignment and multiple alignment: gap insertion penalty 10, gap extension penalty 0.1. An unrooted tree was obtained by cluster analysis of the ITS sequences using the neighbor-joining algorithm with uncorrected distances [38]). Bootstrap support for individual branches was assessed using 1000 replicates. An alternative alignment was performed using the fast Fourier transform method implemented in MAFFT version 5.64 [25]). The most accurate option (L-INS-i) was chosen (iterative refinement method incorporating local pairwise alignments; gap opening penalty: 1.5 and gap extension penalty 0.14).

To estimate a rooted phylogeny, we sequenced two more conserved DNA regions for each ITS haplotype:

• Ca. 530 nucleotides of the nuclear 25S rDNA gene using the primers 25S4R (acaagtgctgagttcctcag; which is specific for *Termitomyces*; [9]) and ITS4R (GCATATCAATAAGCGGAGGA: the reverse complement of the universal primer ITS4; [36]). A region of maximally 11 nucleotides was excluded from the analysis, because it could not be unambiguously aligned.

• Ca. 320 nucleotides of the 12S mitochondrial RNA gene, using the *Termitomyces *specific primer pair ssufw (specific for *Termitomyces*, TCGCGTTAGCATCGTTACTAGT; [9]) and ssurev475 (specific for some *Lyophylleae *including *Termitomyces*, GCCAGAGACGCGAACGTTAGTCG; [9]). A region of maximally 28 nucleotides was excluded from the analysis, because it could not be unambiguously aligned.

The two sequences were manually aligned and analyzed using Bayesian methods. First, the two fungal sequences were tested for combinability using the Partition Homogeneity Test [39] implemented in PAUP*4.0b10 [40], which showed that there was no significant incongruence between the two data sets (1000 artificial data sets, p = 0.14). Bayesian analyses (MrBayes 3.0, [41]) were therefore performed on the combined data set. The two partitions were defined and for each partition a separate model was used (MrModeltest; [42]): SYM + Γ [43]) (lset nst = 6 rates = gamma) for the nuclear 25S and the F81 model + Γ [44]) (lset nst = 1 rates = gamma) for the mitochondrial 12S. Additional maximum parsimony and neighbor-joining analyses (using PAUP*4.0b10 [40]) were performed to check for consistency with the Bayesian results. Sequences have been submitted to Genbank (accession numbers in Table 1 Additional File 1).

### Statistical analysis of host specificity using AMOVA

We used Analysis of Molecular Variance (AMOVA) in Arlequin vers.3.1 [45]) to partition sequence variation among isolates at three hierarchical levels: between host genera, between host species within genera, and between colonies within species. We excluded the 15 allopatric samples from Senegal in the AMOVA analysis, because of the way these were sampled (only one of multiple identical samples was included, so that sampling could be biased). As a measure of genetic distance between ITS haplotypes we used uncorrected pairwise distances. Significance was assessed through 100,000 random permutation replicates. To study the exact source of between host-species symbiont variation, we also performed separate AMOVAs within the two genera with more than a single species, *Odontotermes *and *Microtermes*. Furthermore, we made pairwise comparisons between species, using only the frequency of the different fungal haplotypes of termite species, and not sequence information [27,28]). The rationale behind this was to statistically test the significance of the finding that none of the species in the genus *Odontotermes *shared fungal haplotypes, despite having closely related haplotypes. Since AMOVA takes the genetic distance between the haplotypes into account, it will underestimate differentiation between species in this specific case.

## Authors' contributions

DKA and VIDR carried out the DNA work. DKA and JJB conceived the study. DKA designed and coordinated the study, and analyzed and interpreted the data and drafted the manuscript. DKA, VIDR, HHdFL, CRL, JM, BS and ZWdB contributed with the sampling work and revised the manuscript. JJB revised the manuscript. All authors read and approved the final manuscript.

## Supplementary Material

Additional file 1 Table I. Details of samples used in Aanen et al. Details of samples used in Aanen et al. Click here for file
