# Supplementary material for: Patterns of interaction specificity of fungus-growing termites and Termitomyces symbionts in South Africa
Source: BMC Evol Biol. 2007 Jul 13;7:115. doi: 10.1186/1471-2148-7-115 (PMC1963455; doi:10.1186/1471-2148-7-115)
Supplement: Additional file 1 — Table I. Details of samples used in Aanen et al. Details of samples used in Aanen et al. [file 1471-2148-7-115-S1.doc]

**Online supplementary material Aanen *et al.***

Table I. Details of samples used in Aanen *et al.*

| **Sample nr.** | **Termite genus** | **Termite species** | **Sampling locality** | **Date sampling** | **ITS haplotype fungus** | **ITS Genbank acc.nr.** | **Nuc 25S Genbank acc.nr.** | **Mt 12S**  **Genbank acc.nr.** | **COI Genbank acc.nr.** |
| --- | --- | --- | --- | --- | --- | --- | --- | --- | --- |
| ZA1 | *Macrotermes* | *M. natalensis* | PPRI-farm, Pretoria, South Africa | 28.1.03 | 1 | DQ436938 | EF667071 | EF667086 |  |
| ZA4 | *Macrotermes* | *M. natalensis* | PPRI-farm, Pretoria, South Africa | 28.1.03 | 1 | DQ436938 |  |  | AY818067 |
| ZA8 | *Macrotermes* | *M. natalensis* | Dam, Pietersburg, South Africa | 29.1.03 | 1 | DQ436939 |  |  |  |
| ZA12 | *Macrotermes* | *M. natalensis* | Dam, Pietersburg, South Africa | 29.1.03 | 1 | DQ436940 |  |  |  |
| ZA15 | *Macrotermes* | *M. natalensis* | Dam, Pietersburg, South Africa | 29.1.03 | 1 | DQ436941 |  |  |  |
| ZA33 | *Macrotermes* | *M. natalensis* | PPRI-farm, Pretoria, South Africa | 2.2.03 | 1 | DQ436941 |  |  |  |
| ZA35 | *Macrotermes* | *M. natalensis* | PPRI-farm, Pretoria, South Africa | 2.2.03 | 1 | DQ436942 |  |  |  |
| ZA38 | *Macrotermes* | *M. natalensis* | PPRI-farm, Pretoria, South Africa | 2.2.03 | 1 | DQ436943 |  |  |  |
| ZA39 | *Macrotermes* | *M. natalensis* | PPRI-farm, Pretoria, South Africa | 2.2.03 | 1 | DQ436943 |  |  |  |
| ZA42 | *Macrotermes* | *M. natalensis* | PPRI-farm, Pretoria, South Africa | 2.2.03 | 1 | DQ436944 |  |  |  |
| ZA43 | *Macrotermes* | *M. natalensis* | PPRI-farm, Pretoria, South Africa | 2.2.03 | 1 | DQ436945 |  |  |  |
| ZA107 | *Macrotermes* | *M. natalensis* | PPRI-farm, Pretoria, South Africa | 15.1.04 | 1 | DQ436946 |  |  |  |
| ZA109 | *Macrotermes* | *M. natalensis* | PPRI-farm, Pretoria, South Africa | 16.1.04 | 1 | DQ436947 |  |  |  |
| ZA120 | *Macrotermes* | *M. natalensis* | PPRI-farm, Pretoria, South Africa | 20.1.04 | 1 | DQ436948 |  |  |  |
| ZA123 | *Macrotermes* | *M. natalensis* | PPRI-farm, Pretoria, South Africa | 20.1.04 | 1 | DQ436949 |  |  |  |
| ZA131 | *Macrotermes* | *M. natalensis* | Vygeboomdam, South Africa | 24.1.04 | 1 | DQ436950 |  |  |  |
| ZA133 | *Macrotermes* | *M. natalensis* | Vygeboomdam, South Africa | 24.1.04 | 1 | DQ436951 |  |  |  |
| ZA134 | *Macrotermes* | *M. natalensis* | Vygeboomdam, South Africa | 24.1.04 | 1 | DQ436952 |  |  |  |
| ZA136 | *Macrotermes* | *M. natalensis* | Vygeboomdam, South Africa | 24.1.04 | 1 | DQ436953 |  |  | AY818088 |
| ZA137 | *Macrotermes* | *M. natalensis* | Vygeboomdam, South Africa | 24.1.04 | 1 | DQ436954 |  |  |  |
| ZA139 | *Macrotermes* | *M. natalensis* | Vygeboomdam, South Africa | 24.1.04 | 1 | DQ436955 |  |  |  |
| ZA142 | *Macrotermes* | *M. natalensis* | Vygeboomdam, South Africa | 24.1.04 | 1 | DQ436956 |  |  |  |
| ZA143 | *Macrotermes* | *M. natalensis* | Vygeboomdam, South Africa | 24.1.04 | 1 | DQ436957 |  |  |  |
| ZA144 | *Macrotermes* | *M. natalensis* | Vygeboomdam, South Africa | 24.1.04 | 1 | DQ436958 |  |  |  |
| ZA153 | *Macrotermes* | *M. natalensis* | Close to Amsterdam, South Africa | 25.1.04 | 1 | DQ436959 |  |  |  |
| ZA154 | *Macrotermes* | *M. natalensis* | Close to Amsterdam, South Africa | 25.1.04 | 1 | DQ436960 |  |  |  |
| ZA155 | *Macrotermes* | *M. natalensis* | Close to Amsterdam, South Africa | 25.1.04 | 1 | DQ436961 |  |  |  |
| ZA164 | *Macrotermes* | *M. natalensis* | PPRI-farm, Pretoria, South Africa | 12.2.04 | 1 | DQ436962 |  |  |  |
| ZA166 | *Macrotermes* | *M. natalensis* | PPRI-farm, Pretoria, South Africa | 12.2.04 | 1 | DQ436963 |  |  |  |
| ZA168 | *Macrotermes* | *M. natalensis* | Kwazulu Natal, South Africa | 25.2.04 | 1 | DQ436964 |  |  |  |
| ZA173 | *Macrotermes* | *M. natalensis* | Sabie, South Africa | 4.3.04 | 1 | DQ436965 |  |  |  |
| ZA175 | *Macrotermes* | *M. natalensis* | Edge Kalahari, South Africa | 17.3.04 | 1 | DQ436966 |  |  |  |
|  |  |  |  |  |  |  |  |  |  |
| ZA7 | *Odontotermes* | *O. badius* | PPRI-farm, Pretoria, South Africa | 28.1.03 | 16 | EF636918 | EF667079 | EF667094 | AY818073 |
| ZA19 | *Odontotermes* | *O. badius* | Dam, Pietersburg, South Africa | 29.1.03 | 4 | EF636906 | EF667073 | EF667088 |  |
| ZA108 | *Odontotermes* | *O. badius* | PPRI-farm, Pretoria, South Africa | 15.1.04 | 4 |  |  |  |  |
| ZA157 | *Odontotermes* | *O. badius* | PPRI-farm, Pretoria, South Africa | 4.2.04 | 5 | EF636907 | EF667074 | EF667089 |  |
| ZA158 | *Odontotermes* | *O. badius* | PPRI-farm, Pretoria, South Africa | 4.2.04 | 6 | EF636908 | EF667074 | EF667089 |  |
| ZA159 | *Odontotermes* | *O. badius* | PPRI-farm, Pretoria, South Africa | 4.2.04 | 15 | EF636917 | EF667078 | EF667093 |  |
|  |  |  |  |  |  |  |  |  |  |
| ZA20 | *Odontotermes* | *O. latericius* | PPRI-farm, Pretoria, South Africa | 31.1.03 | 13 | EF636915 | EF667076 | EF667091 |  |
| ZA21 | *Odontotermes* | *O. latericius* | PPRI-farm, Pretoria, South Africa | 31.1.03 | 11 | EF636913 | EF667075 | EF667075 |  |
| ZA22 | *Odontotermes* | *O. latericius* | PPRI-farm, Pretoria, South Africa | 31.1.03 | 18 | EF636920 | EF667080 | EF667095 |  |
| ZA23 | *Odontotermes* | *O. latericius* | PPRI-farm, Pretoria, South Africa | 31.1.03 | 12 | EF636914 | EF667076 | EF667091 |  |
| ZA24 | *Odontotermes* | *O. latericius* | PPRI-farm, Pretoria, South Africa | 31.1.03 | 12 |  |  |  |  |
| ZA26 | *Odontotermes* | *O. latericius* | PPRI-farm, Pretoria, South Africa | 31.1.03 | 12 |  |  |  |  |
| ZA27 | *Odontotermes* | *O. latericius* | PPRI-farm, Pretoria, South Africa | 31.1.03 | 11 |  |  |  |  |
| ZA28 | *Odontotermes* | *O. latericius* | PPRI-farm, Pretoria, South Africa | 31.1.03 | 11 |  |  |  |  |
| ZA50 | *Odontotermes* | *O. latericius* | SABS Farm, Radium, South Africa | 6.2.03 | 10 | EF636912 | EF667075 | EF667075 |  |
| ZA100 | *Odontotermes* | *O. latericius* | PPRI-farm, Pretoria; South-Africa | 14.1.04 | 12 |  |  |  |  |
| ZA102 | *Odontotermes* | *O. latericius* | PPRI-farm, Pretoria, South Africa | 14.1.04 | 12 |  |  |  |  |
| ZA124 | *Odontotermes* | *O. latericius* | PPRI-farm, Pretoria, South Africa | 20.1.04 | 11 |  |  |  |  |
| ZA128 | *Odontotermes* | *O. latericius* | PPRI-farm, Pretoria, South Africa | 22.1.04 | 12 |  |  |  |  |
| ZA160 | *Odontotermes* | *O. latericius* | PPRI-farm, Pretoria, South Africa | 4.2.04 | 11 |  |  |  |  |
| ZA162 | *Odontotermes* | *O. latericius* | PPRI-farm, Pretoria, South Africa | 4.2.04 | 11 |  |  |  |  |
| dka13 | *Odontotermes* | *O. latericius* | Kolda, Senegal | 4.12.00 | 19 | EF636921 |  |  |  |
| dka15 | *Odontotermes* | *O. latericius* | Kolda, Senegal | 4.12.00 | 9 | EF636911 | EF667075 | EF667075 |  |
| dka20 | *Odontotermes* | *O. latericius* | Kolda, Senegal | 4.12.00 | 2 | EF636904 | AY127785 | AY127836 |  |
| dka24 | *Odontotermes* | *O. latericius* | Kolda, Senegal | 4.12.00 | 9 |  |  |  |  |
| dka28 | *Odontotermes* | *O. latericius* | Kolda, Senegal | 5.12.00 | 8 | EF636910 | EF667075 | EF667075 |  |
| dka29 | *Odontotermes* | *O. latericius* | Kolda, Senegal | 5.12.00 | 2 |  |  |  |  |
| dka39 | *Odontotermes* | *O. latericius* | Kolda, Senegal | 5.12.00 | 9 |  | AY127794 | AY127752 |  |
| dka51 | *Odontotermes* | *O. latericius* | Kolda, Senegal | 5.12.00 | 19 |  |  |  |  |
| dka82 | *Odontotermes* | *O. latericius* | Tambacounda, Senegal | 7.12.00 | 2 |  |  |  |  |
| dka84 | *Odontotermes* | *O. latericius* | Tambacounda, Senegal | 7.12.00 | 17 | EF636919 |  |  |  |
| dka85 | *Odontotermes* | *O. latericius* | Tambacounda, Senegal | 7.12.00 | 2 |  |  |  |  |
| dka90 | *Odontotermes* | *O. latericius* | Tambacounda, Senegal | 7.12.00 | 17 |  |  |  |  |
| dka92 | *Odontotermes* | *O. latericius* | Tambacounda, Senegal | 7.12.00 | 17 |  |  |  |  |
| dka93 | *Odontotermes* | *O. latericius* | Tambacounda, Senegal | 7.12.00 | 17 |  | AY127767 | AY127851 |  |
|  |  |  |  |  |  |  |  |  |  |
| ZA17 | *Odontotermes* | *O. transvaalensis* | Dam, Pietersburg, South Africa | 29.1.03 | 7 | EF636909 | EF667074 | EF667089 | AY818076 |
| ZA51 | *Odontotermes* | *O. transvaalensis* | SABS Farm, Radium, South Africa | 6.2.03 | 3 | EF636905 | EF667072 | EF667087 |  |
| ZA53 | *Odontotermes* | *O. transvaalensis* | SABS Farm, Radium, South Africa | 6.2.03 | 3 |  |  |  |  |
| ZA54 | *Odontotermes* | *O. transvaalensis* | SABS Farm, Radium, South Africa | 6.2.03 | 3 |  |  |  |  |
| ZA55 | *Odontotermes* | *O. transvaalensis* | SABS Farm, Radium, South Africa | 6.2.03 | 14 | EF636916 | ÈF667077 | EF667092 |  |
|  |  |  |  |  |  |  |  |  |  |
| ZA5 | *Microtermes* | M. sp. I | PPRI-farm, Pretoria, South Africa | 28.1.03 | 20 | EF636922 | EF667081 | EF667098 |  |
| ZA9 | *Microtermes* | M. sp. I | Dam, Pietersburg, South Africa | 29.1.03 | 20 |  |  |  | AY818069 |
| ZA25 | *Microtermes* | M. sp. I | PPRI-farm, Pretoria, South Africa | 31.1.03 | 24 | EF636926 | EF667084 | EF667099 |  |
| ZA31 | *Microtermes* | M. sp. I | PPRI-farm, Pretoria, South Africa | 31.1.03 | 24 |  |  |  | AY818074 |
| ZA34 | *Microtermes* | M. sp. I | PPRI-farm, Pretoria, South Africa | 2.2.03 | 23 | EF636925 | EF667084 | EF667099 |  |
| ZA36 | *Microtermes* | M. sp. I | PPRI-farm, Pretoria, South Africa | 2.2.03 | 24 |  |  |  |  |
| ZA37 | *Microtermes* | M. sp. I | PPRI-farm, Pretoria, South Africa | 2.2.03 | 24 |  |  |  |  |
| ZA40 | *Microtermes* | M. sp. I | PPRI-farm, Pretoria, South Africa | 2.2.03 | 23 |  |  |  |  |
| ZA41 | *Microtermes* | M. sp. I | PPRI-farm, Pretoria, South Africa | 2.2.03 | 20 |  |  |  |  |
| ZA70 | *Microtermes* | M. sp. I | Pietermaritzburg, South Africa | 18.2.02 | 24 |  |  |  |  |
| ZA101 | *Microtermes* | M. sp. I | PPRI-farm, Pretoria, South Africa | 14.1.04 | 25 | EF636927 | EF667085 | EF667100 |  |
| ZA112 | *Microtermes* | M. sp. I | Close to Pretoria, South Africa | 16.1.04 | 24 |  |  |  |  |
| ZA122 | *Microtermes* | M. sp. I | PPRI-farm, Pretoria, South Africa | 20.1.04 | 24 |  |  |  |  |
| ZA126 | *Microtermes* | M. sp. I | PPRI-farm, Pretoria, South Africa | 22.1.04 | 22 | EF636924 | EF667083 | EF667098 |  |
| ZA129 | *Microtermes* | M. sp. I | PPRI-farm, Pretoria, South Africa | 22.1.04 | 24 |  |  |  |  |
| ZA165 | *Microtermes* | M. sp. I | PPRI-farm, Pretoria, South Africa | 12.2.04 | 25 |  |  |  |  |
| ZA167 | *Microtermes* | M. sp. I | PPRI-farm, Pretoria, South Africa | 12.2.04 | 24 |  |  |  |  |
| ZA169 | *Microtermes* | M. sp. I | Kwazulu Natal, South Africa | 25.2.04 | 25 |  |  |  |  |
|  |  |  |  |  |  |  |  |  |  |
| ZA138 | *Microtermes* | M. sp. II | Vygeboomdam, South Africa | 24.1.04 | 21 | EF636923 | EF667082 | EF667097 | AY818089 |
| ZA141 | *Microtermes* | M. sp. II | Vygeboomdam, South Africa | 24.1.04 | 25 |  |  |  |  |
|  |  |  |  |  |  |  |  |  |  |
| ZA10 | *Microtermes* | M. sp. III | Dam, Pietersburg, South Africa | 29.1.03 | 24 |  |  |  | AY818071 |
| ZA48 | *Microtermes* | M. sp. III | SABS Farm, Radium, South Africa | 6.2.03 | 24 |  |  |  |  |
| ZA132 | *Microtermes* | M. sp. III | Vygeboomdam, South Africa | 24.1.04 | 25 |  |  |  |  |
| ZA135 | *Microtermes* | M. sp. III | Vygeboomdam, South Africa | 24.1.04 | 25 |  |  |  | AY818087 |
| ZA140 | *Microtermes* | M. sp. III | Vygeboomdam, South Africa | 24.1.04 | 24 |  |  |  |  |
| ZA145 | *Microtermes* | M. sp. III | Vygeboomdam, South Africa | 24.1.04 | 25 |  |  |  |  |
|  |  |  |  |  |  |  |  |  |  |
| ZA49 | *Microtermes* | M. sp. IV | SABS Farm, Radium, South Africa | 6.2.03 | 21 |  |  |  | AY818075 |
| ZA52 | *Microtermes* | M. sp. IV | SABS Farm, Radium, South Africa | 6.2.03 | 20 |  |  |  |  |
|  |  |  |  |  |  |  |  |  |  |
| ZA161 | *Microtermes* | ? | PPRI-farm, Pretoria, South Africa | 4.2.04 | 24 |  |  |  |  |
